# Muti-Frame Point Cloud Feature Fusion Based on Attention Mechanisms for 3D Object Detection

**DOI:** 10.3390/s22197473

**Published:** 2022-10-02

**Authors:** Zhenyu Zhai, Qiantong Wang, Zongxu Pan, Zhentong Gao, Wenlong Hu

**Affiliations:** 1 Aerospace Information Research Institute, Chinese Academy of Sciences, Beijing 100190, China; 2 Key Laboratory of Technology in Geo-Spatial Information Processing and Application System, Chinese Academy of Sciences, Beijing 100190, China; 3 School of Electronic, Electrical and Communication Engineering, University of Chinese Academy of Sciences, Beijing 101408, China

**Keywords:** autonomous driving, 3D object detection, point cloud sequences, attention mechanism, feature fusion

## Abstract

Continuous frames of point-cloud-based object detection is a new research direction. Currently, most research studies fuse multi-frame point clouds using concatenation-based methods. The method aligns different frames by using information on GPS, IMU, etc. However, this fusion method can only align static objects and not moving objects. In this paper, we proposed a non-local-based multi-scale feature fusion method, which can handle both moving and static objects without GPS- and IMU-based registrations. Considering that non-local methods are resource-consuming, we proposed a novel simplified non-local block based on the sparsity of the point cloud. By filtering out empty units, memory consumption decreased by 99.93%. In addition, triple attention is adopted to enhance the key information on the object and suppresses background noise, further benefiting non-local-based feature fusion methods. Finally, we verify the method based on PointPillars and CenterPoint. Experimental results show that the mAP of the proposed method improved by 3.9% and 4.1% in mAP compared with concatenation-based fusion modules, PointPillars-2 and CenterPoint-2, respectively. In addition, the proposed network outperforms powerful 3D-VID by 1.2% in mAP.

## 1. Introduction

In dense point cloud scenes, the geometric shape of the object is relatively complete. However, these lidar techniques, which use more laser beams, are expensive as well. Reducing the cost of lidar techniques is a problem in the large-scale application of automatic driving. The autopilot company nuTonomy tried to use cheap 32-line lidar and released the NuScenes dataset [1]. Unlike the KITTI dataset [2], which uses 64-line lidar, the NuScenes dataset is built with 32-line lidar, exacerbating the sparsity of point clouds. Therefore, NuScenes officially recommends concatenating 10 calibrated point cloud frames to obtain denser point clouds. Compared with single-frame point cloud, multiple frames provide a denser description of the surrounding environment as a result of multi-view observations.

**Figure 1:**
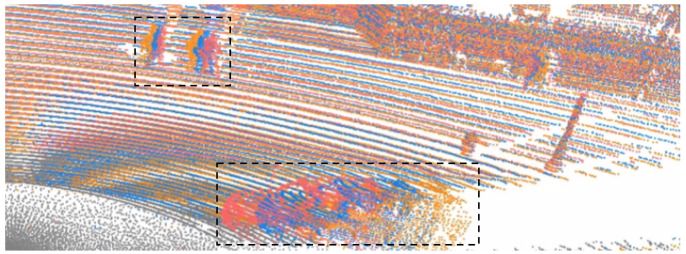
Multiple frames are concatenated into one frame by registration. The black dashed box marks the area where motion blur occurs.

Currently, multi-frame-based object detectors inevitably face the problem of registration between different frames. Usually, most of them align different frames via GPS and IMU, etc. [3,4,5,6,7]. However, registration can align static objects but not moving objects. Consequently, such fusion will cause motion blur [3,8]. As shown in Figure 1, motion blur emerges around a fast-moving car and walking humans. Wenjie Luo et al. [5] use “shadow” to describe the motion blur of objects. They believe “shadow” represents the motion state of objects, and it assists in motion forecasting. However, for object detection, the distorted object’s shape will result in inaccurate detection results. To alleviate the problem, 3D-VID [8] uses deformable convolutions [9] to align fast-moving objects on the basis of ego-motion transformation. SDP-Net [10] uses scene flows to align moving and static objects. However, this alignment method depends on the prediction accuracy of the scene’s flow module.

To solve the above problems, we proposed a novel multi-frame fusion strategy that can align not only static objects but also moving objects without external information. The proposed strategy applies non-local networks [11] to fuse multiple frames at the feature level. The non-local-based module fuses multiple frames by calculating similarities between features from different frames. However, the calculation consumes massive computing resources. To reduce resource consumption, we designed a non-local module with an index table, which is referred to as index-nonlocal. In addition, some targets are highly similar to the background, which affects the similarity calculation between features. We apply triple attention (TANet) [12] to enhance the key information of the object and suppress background noise. Finally, we implement our method based on anchor-based and anchor-free detectors, and verified the performance of the model on the NuScenes dataset. Experiments show that the proposed method outperforms multi-frame concatenation-based baseline models and exceeds the strong multi-frame model 3D-VID by 1.2% in mAP.

Our main contributions can be summarized as follows:

- We propose a method by applying the non-local network to fuse two-frame point clouds. This method does not need external-information-based registration and can handle stationary and moving objects.
- To solve the problem that non-local-based fusion modes consume massive computing resources, we propose a non-local network with an index table, which only calculates similarities among non-empty units.
- We apply the triple attention mechanism to suppress the background noise and enhance the key information. It plays a role in improving the performance of the non-local fusion module.
- The proposed method is universal on grid-based lidar detectors and can be easily migrated. In this paper, we verify the method based on PointPillars [13] and CenterPoint [14].

## 2. Related Work

Generally, 3D object detection methods can be divided into three categories: 2D image-based methods [15,16,17,18], point-cloud-based methods [3,5,8,13,14,19,20,21,22,23,24], and multi-sensor fusion-based methods [25,26,27,28,29]. In this article, we mainly focus on point-cloud-based methods. According to the number of point cloud frames used by detectors, the existing point-cloud-based detectors can be classified as single-frame-based detectors and multi-frame-based detectors.

### 2.1. Single-Frame-Based Lidar Object Detection

Main single-frame methods can be classified into point-based methods [19,22,23] and grid-based methods [13,14,20,21].

For point-based methods, PointNet [22] directly takes raw point clouds as input. Then, it learns points features through several MLP layers. Finally, it learns global features through max pooling. As PointNet extracts feature from single point, it cannot describe contextual information. PointNet++ [23] adds a multi-level feature extraction structure based on PointNet, which enhances the description ability of fine local geometric structures. VoteNet [30] utilizes PointNet++ as the backbone network and designs a voting mechanism to detect objects. PointRCNN [19] also uses PointNet++ as the backbone network to build a two-stage network, which further improves the accuracy of object detection. Point-based methods require point-wise operations, so it is hard to meet the real-time requirements of autonomous driving when the number of points is large.

Grid-based methods perform better in efficiency due to the fact that they divide raw point clouds into regular grids. Those methods include VoxelNet [21], SECOND [20], PointPillars [13], and CenterPoint [14], etc. VoxelNet divides the point cloud into regular voxel grids. Then, it uses the idea of PointNet to extract voxel features to form pseudo-images. Finally, the method extracts features through 3D convolutions. SECOND follows the network framework of VoxelNet. It proposes to apply sparse convolution to extract point cloud features, which significantly improves the training and inference speed of the network. Different from VoxelNet and SECOND, which divide point clouds into voxels, PointPillars divides point clouds into pillars. In detail, pillar-based voxelization only results in discrete point clouds in the horizontal plane. In addition, the method replaces 3D convolution with 2D convolution. The above two improvements greatly accelerated the model’s running speed. This is why PointPillars is widely applied in the autonomous-driving industry. Recently, CenterPoint introduced an anchor-free detection head in 3D object detection and has achieved remarkable performance improvements.

### 2.2. Multi-Frame-Based Lidar Object Detection

In recent years, more scholars began to study the object detection method based on multiple frames point cloud [3,4,5,8,10,24,31]. FaF [5] concatenates five aligned frames as input and performs detection, tracking, and motion forecasting in one framework. SDP-Net [10] uses scene flows to align multiple frame features and fuse them via different weights. WYSIWYG [24] proposes to concatenate multiple frames into a single frame to expand the visibility area of the current frame. Both YOLO4D [4] and Unet-LSTM [3] apply LSTM networks to utilize the spatiotemporal information in point cloud sequences. The method of 3D-VID [8] first studies object detection from the perspective of 3D point cloud videos. The method applies graph neural networks [32] and convGRU networks [33] to utilize the spatiotemporal information. To solve the problem of motion blur, ego-motion information is introduced to register different frames, and deformable convolution [9] is used to align moving objects.

### 2.3. Attention Mechanism

Attention mechanisms are widely used in various fields of deep learning and have resulted in various types. The essence of the attention mechanism is to imitate human selective visual attention. In detail, the attention mechanism emphasizes key information by dynamic weighting. Self-attention mechanisms are one of the attention mechanisms. A non-local network [11], which is used in this work, is a self-attention mechanism. It can capture long-range dependence well. Therefore, it has the ability to establish a relationship between different frames. One of its defects is that it needs massive memory resources. Various methods are designed to reduce the space complexity of non-local networks. CCNet [34] split non-local networks into row-wise and column-wise self-attention. Then, two consecutive sparse self-attention mechanisms are used to approximate one dense self-attention calculation. LRNet [35] confines the self-attention calculation in a local area, not in the global area. ANN [36] utilizes pyramid pooling to reduce the space the complexity of the non-local network. ISSNet [37] factorizes the dense correlation matrix into the product of two sparse correlation matrices, which greatly reduces the complexity of time and space. DGMN [38] abstracts the feature map into a graph structure and designs a dynamic graph message-passing network. The space complexity of self-attention computation is greatly reduced. Recently, Swin-transformer [39] limits the computation of self-attention to a local window, reducing computation resources.

## 3. Methods

In this section, we first present the overall framework of our method in Section 3.1. Then, we introduce some pre-operations of 3D object detection in Section 3.2. Then, a multi-frame fusion method and the index-nonlocal model are illustrated in Section 3.3 and Section 3.4. Afterward, we introduce a method for using triple attention (TANet) to improve non-local module performances in Section 3.5. Finally, we provide more details on our framework in Section 4.2.

### 3.1. Overview

**Figure 2:**
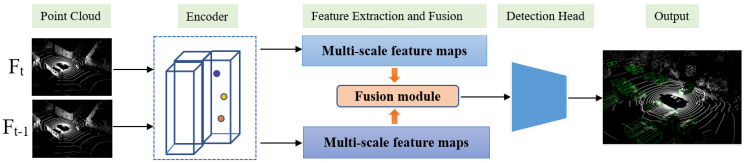
The overall framework of our proposed multi-frame fusion method.

As shown in Figure 2, the framework mainly includes three parts: point cloud encoder, feature extraction and fusion, and detection head. First, two adjacent point cloud frames are encoded to form 2D pseudo-images. Next, two adjacent frames are fed into the same feature extraction network. Then, the non-local-based fusion module, modeling the relationship between objects within two frames, was adopted to fuse feature maps of two frames. Finally, fused feature maps are fed into the detection head. In this paper, we implement the method on the anchor-based networks: PointPillars and anchor-free network CenterPoint.

### 3.2. Grid-Based Point Cloud Encoder

Lidar continuously senses the surrounding environment by emitting laser beams, and one frame point cloud $F_{t}$ is generated at each time step *t*. Each point $P_{i}$ in the frame is represented by {x,y,z,r}, in which (x,y,z) and *r* represent location coordinates and reflection intensities, respectively. A frame of point cloud $F_{t}$ is composed of point set $\left\{P_{1},P_{2},P_{3},...,P_{i}\right\}$, and there is no fixed order among points.

Due to the disorder and irregularity of point clouds, the 2D convolution network (2D CNN) cannot be applied to extract features. To produce a point cloud with a structure suitable for 2D CNN, a grid-based point cloud encoder is used to generate a regular pseudo-image. Generally, there are two grid-based voxelization forms: voxel-based voxelization and pillar-based voxelization. By comparison, voxel-based voxelization discretes the point cloud in the *x*, *y*, and *z* axes; Pillar-based voxelization discretes the point cloud in the *x* and *y* axes. In this paper, pillar-based voxelization is adopted to generate pseudo images.

**Figure 3:**
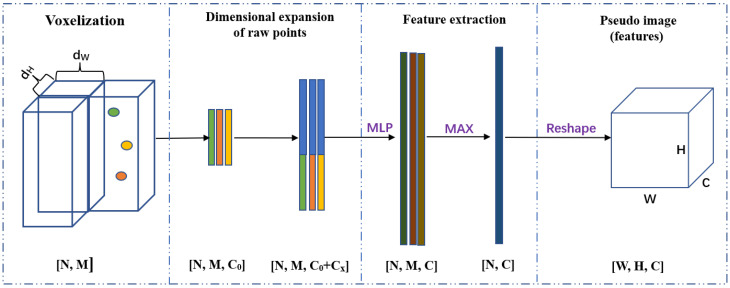
The overall process of grid-based point cloud encoder.

As shown in Figure 3, the grid-based point cloud encoder contains four parts: voxelization, dimensional expansion, feature extraction, and pseudo image generation. First, each frame of the point cloud is divided into *N* pillars, and each pillar retains *M* points. If the number of points is less than *M* in one pillar, use zero-point to fill the pillar. Second, the encoder appends the geometric center $\left(x_{c},y_{c},z_{c}\right)$ and the arithmetic mean center $\left(x_{m},y_{m},z_{m}\right)$ of the pillar to each point as new channels. After dimensional expansion, all point channels are expended from $C_{0}$ to $\left(C_{0}+C_{x}\right)$. Third, all points in each pillar are fed to the feature extraction module, which consists of a fully connected layer (MLP) and max pooling layers. Finally, each pillar is placed in its original position by reshaping. Then, a pseudo image with the shape of [W,H,C] is generated, and 2D CNN can be used in the following steps.

### 3.3. Feature Fusion

**Figure 4:**
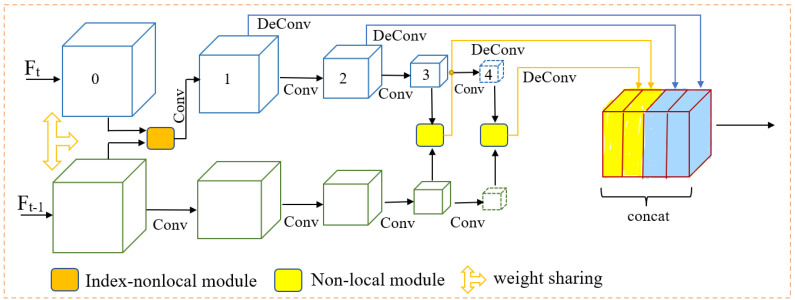
Feature extraction and fusion network. The 0th layer is the pseudo-image, which is generated by the point cloud encoder.

After the grid-based point cloud encoder, each frame is fed to a classic multi-scale feature extractor. Then, several non-local-based modules are used to fuse feature maps. As shown in Figure 4, the backbone of our method can be divided into two parts: feature extraction module and non-local-based fusion module. The feature extraction module includes two branches sharing the same weights. The upper branch processes frame $F_{t}$, and the lower branch processes frame $F_{t-1}$.

The non-local-based fusion module is used to fuse multi-scale features. Then, fused features are concatenated and fed into the detection head. It must be mentioned that the 0th layer feature map is the pseudo-image generated by the encoder. It is also a high-resolution feature map that contains rich spatial information.

**Figure 5:**
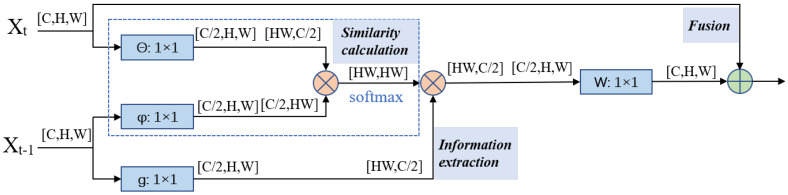
Non-local module. The blue symbols represent 1 × 1 convolutions, the orange symbols represent matrix multiplication, and the green symbols represent element-wise addition.

The non-local module can capture long-range dependence, so it can be used to establish a relationship between two regions in the image or different frames in the video. This paper uses the non-local module to fuse two adjacent point cloud frames at the feature level. As shown in Figure 5, the non-local fusion module has three stages: similarity calculation, information extraction, and fusion. First, the feature maps of two frames access θ and φ branches to calculate the similarity. After the normalization operation, the correlation matrix between the two frames is obtained. It contains similarity information and relative position relationships between the pixels of two feature maps. Then, the correlation matrix is used to extract information from the $F_{t-1}$. Finally, the extracted information is element-wise added to the feature map of $F_{t}$.

The non-local module is particularly useful for establishing a connection between two adjacent frames, but it is criticized for its vast memory consumption. Applying non-local modules in the low-resolution feature maps is affordable, but the amount of resource consumption is unaffordable for high-resolution feature maps. Hence, we adopt two different schema. As shown in Figure 4, for low-resolution feature maps, we directly adopt the non-local module. For high-resolution feature maps, we propose a simplified non-local module.

### 3.4. Index-Nonlocal Module

In this subsection, we first reveal which step dominates the computation by profoundly analyzing the calculation process. Then, a novel simplified method that utilizes the point cloud’s unique property will be introduced.

#### 3.4.1. Analysis of Non-local Calculation

The fusion module is shown in Figure 5. It is proposed based on the classical non-local module. The input of classical non-local is one image. By contrast, the input of the fusion module is two adjacent feature maps $X_{t},X_{t-1}\in R^{C\times H\times W}$. There are three 1×1 convolutions: $W_{\theta }$, $W_{\varphi }$, and $W_{g}$ are used to transform $X_{t}$ and $X_{t-1}$ for embedding. It can be illustrated as follows.

(1) $$\theta =W_{\theta }\left(X_{t}\right),\;\varphi =W_{\varphi }\left(X_{t-1}\right),\;g=W_{g}\left(X_{t-1}\right),\;\theta ,\varphi ,g\in R^{C^{\prime }\times H\times W}$$

After that, the feature size is flattened to $C^{\prime }\times N$, where N=H×W. Then, the correlation matrix $U\in R^{N\times N}$ is calculated by matrix multiplication.

(2) $$U=\varphi^{T}\times \theta ,\;U\in R^{N\times N}$$

Next, softmax is used to normalize the correlation matrix row-by-row.

(3) $$\bar{U}=softmax\left(U\right)$$

Afterward, correlation matrix $\bar{U}$, which contains spatial location and similarity weight information, is used to extract features from $X_{t-1}$.

(4) $$V=\bar{U}\times g^{T},\;V\in R^{N\times C^{\prime }}$$

Finally, extracted features are added to $X_{t}$:

(5) $$Out=W_{o}\left(V^{T}\right)+X_{t},\;Out\in R^{C\times H\times W}$$

where $W_{o}$ is also a 1×1 convolution, which is used to recover the feature channel’s dimension from $C^{\prime }$ to *C*.

From the above analysis, it can be clearly observed that Equations (2) and (4) dominate the computation process. The space complexity of the two matrix multiplication is both $O\left(C^{\prime }N^{2}\right)=O\left(C^{\prime }H^{2}W^{2}\right)$. It can be observed that the large matrix multiplication consumes most of memory resources. In this paper, the pseudo image shape is 64×512×512, that is, $N^{2}={\left(512\times 512\right)}^{2}=68,719,476,736$. Therefore, the memory occupation can be computed as follows: $Space=\frac{C}{2}\times N^{2}\times size\;of\left(float32\right)=8192$ GB. It is can be seen that the module consumes massive space resources.

The above analysis can be summarized as follows.

(6) $$Equation\;\left(2\right):R^{N\times C^{\prime }}\times R^{C^{\prime }\times N}⟶Equation\;\left(4\right):R^{N\times N}\times R^{N\times C^{\prime }}⟶R^{N\times C^{\prime }}$$

Hence, size *N* of the image directly determines the calculation scale of the non-local module. Motivated by ANN [36], we considered whether *N* can be reduced by sampling. Unlike the pyramid pooling sampling method adopted by ANN, we propose a sampling strategy based on the sparsity of point clouds.

#### 3.4.2. Simplify Non-local Modules with an Index Table

As mentioned in Section 3.2, point clouds are different from 2D images. Two properties of the point cloud are used to simplify non-local modules. First, the sampling points of lidar only occupy a small part of the 3D space. We used statistics in the average proportion of empty pillars in each frame in the NuScenes dataset. The result is that the proportion of empty pillars is 97.29%. Second, the non-empty pillars’ coordinate can be obtained in the voxelization stage. That is a huge difference compared with natural images. In natural images with sparse objects, the coordinate of empty pixels can not be directly obtained. Nevertheless, each point of point clouds has an accurate and unique coordinate. In the encoder, these points are divided into different pillars by referring to coordinates. Hence, the accurate coordinate of non-empty pillars can be easily obtained. In short, the coordinate index table can be used as a guide for sampling.

Based on the two properties, we propose three sampling blocks, $P_{\theta }$, $P_{\varphi }$, and $P_{g}$, after θ, φ, and *g*. This can be described as follows.

(7) $$\theta_{p}=P_{\theta }\left(\theta \right),\;\varphi_{p}=P_{\varphi }\left(\varphi \right),\;g_{p}=P_{g}\left(g\right),\;\theta_{p}\in R^{S_{t}},\varphi_{p},P_{g}\in R^{S_{t-1}}$$

Then, the correlation matrix is calculated by the following.

(8) $$U_{p}=\varphi_{p}^{T}\times \theta_{p},\;U_{p}\in R^{S_{t}\times S_{t-1}}$$

Next, the normalized correlation matrix is used to extract features from $X_{t-1}$.

(9) $$V_{p}={\bar{U}}_{p}\times {g_{p}}^{T},\;V_{p}\in R^{S_{t}\times C^{\prime }}$$

After that, we scatter these key pixel points into their original position. The blank area is kept at zero, similarly to original feature maps. Next, the normalized correlation matrix is used to extract features from $X_{t-1}$.

(10) $$V_{sc}=Scatter\left(V_{p}\right),\;V_{sc}\in R^{\hat{N}\times C^{\prime }}$$

Finally, fused features are obtained by the following equation.

(11) $$Out=W_{o}\left({V_{sc}}^{T}\right)+X_{t},\;Out\in R^{C\times H\times W}$$

In the index-nonlocal module, blank areas are filtered out by the index table while only key feature points are kept. The new matrix multiplications can be illustrated as follows.

(12) $$Equation\;\left(2\right):R^{S_{t}\times C^{\prime }}\times R^{C^{\prime }\times S_{t-1}}⟶Equation\;\left(4\right):R^{S_{t}\times S_{t-1}}\times R^{S_{t-1}\times C^{\prime }}⟶R^{\hat{N}\times C^{\prime }}$$

**Figure 6:**
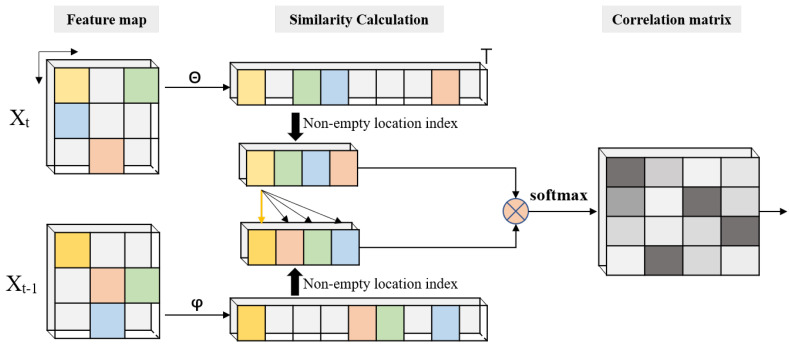
The correlation matrix calculation of index-nonlocal module. In the feature map and similarity calculation stage, grids with color represent non-empty units. The classes of color represent the similarity of feature points. In the correlation matrix, the gray level represents the relevance among feature points.

As shown in Equation (12), the space complexity of matrix multiplication in the index-nonlocal is only $O(C^{\prime }S^{2})$, which is obviously lower than $O\left(C^{\prime }N^{2}\right)\;\left(S\approx 0.0271\times N\right)$ of the original version. As shown in Equation (13), by filtering out empty units, the memory consumption is decreased by 99.93%. The matrix calculation process is shown in Figure 6; non-empty feature points are selected with the non-empty location index table. Then, the correlation matrix is obtained by pixel-wise multiply and row-wise normalizing.

(13) $$\frac{N^{2}-{\left(0.0271N\right)}^{2}}{N^{2}}\times 100\%\approx 99.93\%$$

After being simplified, the fusion module can be used in high-resolution feature maps with rich spatial information. As mentioned in Section 3.3, the index-nonlocal is only added in the 0th layer of multi-scale feature maps. The index-nonlocal block is hard to use in other layers, because the coordinate index table cannot be accurately mapped to the feature map of other scales by a simple linear transformation. Hence, the index-nonlocal is only used in the 0th layer.

### 3.5. Point Cloud Triple Attention Mechanism

In the scheme, the non-local-based fusion module is a self-attention mechanism. On the one hand, the fusion effect of the module depends on the ability of the detector’s feature extraction. Inaccurate features will lead to an unreliable correlation matrix between the two frames. Furthermore, the unreliable correlation matrix will weaken key features after fusion. On the other hand, small objects or distant objects that have few valid scanned points are highly similar to the background. For example, in complex scenes, it is hard to distinguish pedestrians from the background (e.g., trees, bushes, and poles). Those background objects are easily associated with foreground objects. Hence, it is necessary for the proposed method to increase the distance between features of foreground objects and background before fusion.

**Figure 7:**
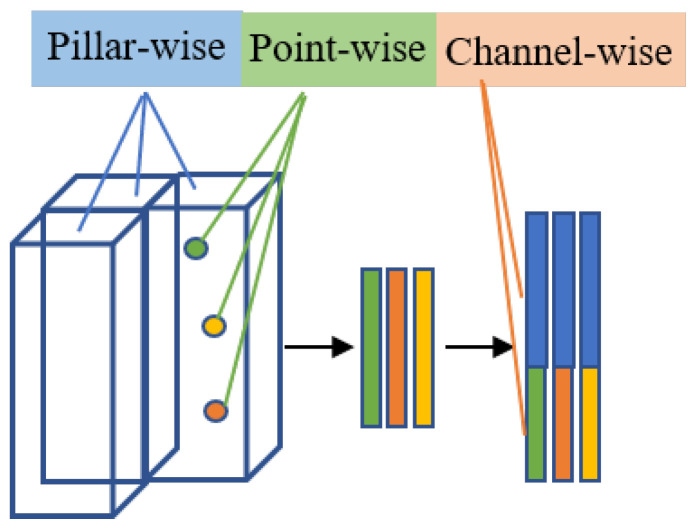
The position in which triple attention is applied.

In this paper, the triple attention (TANet) [12] is adopted to enhance crucial information and suppress background noise. As shown in Figure 7, the TANet module is used between the voxelization and feature extraction in the encoder. Similarly to SENet [40], TANet highlights essential points, channels, and pillars by combining point-wise attention, channel-wise attention, and pillar-wise attention. Point-wise and channel-wise are used to judge the importance of each point and each channel in the pillars. Pillar-wise attention is used to judge the importance of each pillar in all pillars. After that, the likelihood of foreground objects being associated with the background is greatly reduced. In short, TANet enhances the reliability of the fusion module by suppressing irrelevant features and enhancing key features.

## 4. Experiments

In this section, we first briefly introduce the dataset used in the experiment. Then, more implementation details are provided in Section 4.2. Finally, experimental results and analysis are presented in Section 4.3.

### 4.1. Dataset

In this study, the NuScenes dataset, which provides point cloud sequences, is chosen to validate the proposed method. The sampling frequency of lidar is 20 Hz. The dataset consists of 1000 driving scenarios, each lasting 20 s. The dataset includes 10 classes of objects, namely car, pedestrian, bus, barrier, traffic cone, truck, trailer, motorcycle, construction vehicle, and bicycle. We follow the official standard for the division of training sets, validation sets, and test sets.

**Figure 8:**
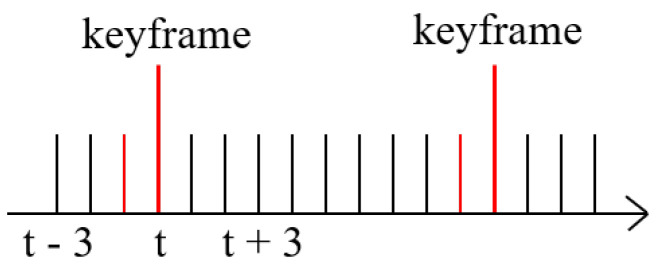
The relationship between keyframes and intermediate frames. The red line represents the point cloud frames used in this study.

The dataset extracts and labels one frame every 0.5 s. These frames with annotations are called keyframes, while the remaining unlabeled frames are called intermediate frames. As shown in Figure 8, there are nine intermediate frames between every two keyframes.

### 4.2. Implementation Details

We reimplement PointPillars and CenterPoint as our backbone network with reference to [13,14,41,42]. To verify the effect of fusing multi-frames of raw point cloud, based on PointPillars, we carried out groups of experiments: 1. feed a single frame of point cloud into the PointPillars; 2. fuse two aligned frames of point clouds, $F_{t}$ and $F_{t-1}$, as one frame and feed it into the PointPillars; 3. fuse two unaligned frames of point cloud, $F_{t}$ and $F_{t-1}$, as one frame and feed it into the PointPillars. All networks are trained with correspond data.

In this study, we design a multi-frame based lidar detector: MFFFNet (multi-frame feature fusion network). As mentioned in Section 3, the input of the proposed method is two adjacent frames $F_{t}$ and $F_{t-1}$. Then, the backbone network (PointPillars or CenterPoint) is used to extract features of the two frames, respectively. After that, the non-local-based module is used to fuse multi-scale features. Frame $F_{t-1}$ is used to provide additional information for frame $F_{t}$. The network uses the ground truth provided by keyframe $F_{t}$.

For data augmentation, we use the same augmentation method as PointPillars. It includes a random insertion of ground truth, random flips along the *x* and *y* axes, random global rotation, and random global scaling. The two adjacent frames perform data augmentation synchronously, and frame $F_{t-1}$ follows the parameters of frame $F_{t}$.

In the above experiments, the range of *x*, *y*, and *z* is ([−51.2,51.2], [−51.2,51.2], and [−5.0,3.0]) meters, respectively. The size of each pillar is [0.2,0.2,8] meters, so the number of pillars per frame is N(N=262,144). In addition, the number of points in each pillar is M(M=20). The model is trained on a GPU (NVIDIA TESLA V100 32G) for 80 epochs with a batch size of 4. We use the Adam optimizer and the one-cycle strategy with an initial learning rate of 0.001. We follow the metric used in almost all autopilot datasets, which is the mean average precision (mAP).

### 4.3. Results

In this part, experiment results are presented to verify the proposed method. In addition, experiment results also show that the proposed method has significant advantages over other multi-frame methods.

#### 4.3.1. Quantitative Analysis

In this subsection, we first verify that multi-frame fusion benefits the performance of object detectors. Then, the importance of registration for concatenation-based fusion methods is verified. After that, the performance of the proposed fusion method and the concatenation-based fusion method is compared. Finally, a comparison between different 3D detectors on the NuScenes dataset is illustrated.

As shown in Table 1, the mAP of PointPillars-2 is 2.0% higher than PointPillars-1. This is because concatenating multiple frames can obtain a denser point cloud of the surrounding environment. The dense point cloud means that it contains more geometric details of objects. As the geometric shape of objects is relatively complete, it can provide richer structure information. Hence, the high-resolution point cloud is a benefit for the performance improvement of the detector.

As shown in Table 2, inputting two adjacent frames without registration into the PointPillars-2 leads to the mAP drop by 2.1%. Its performance is even lower than inputting one frame into PointPillars-1. Concatenating two frames without registration means that the influence of ego-motion is not eliminated. Hence, concatenating multiple frames without registration will cause a misalignment of objects. The misaligned objects have distortion geometry shape, seriously interfering with feature extraction. Inaccurate features will lead to erroneous results. Therefore, registration is essential for the concatenation-based method.

Although concatenating multiple frames can improve the detector’s performance, the concatenation-based fusion method must use external information for registration. In additon, it can not align moving objects. Thus, this fusion method is not a highly efficient method.

To solve the above problems, we propose a non-local-based fusion method that does not need registration. As shown in Table 3, our fusion method comprehensively outperforms two networks with the same number of input frames. The experiment on PointPillars-2 shows that our method brings a performance improvement of 3.9% in mAP. Among all categories, barrier and construction vehicle (CV) have the most remarkable improvement, increasing 6.8% in AP. The experiment on CenterPoint-2 also shows an improvement in all categories with an increase of 4.1% in mAP.

The proposed method, which fuses two frames by calculating similarity in the feature level, does not require registration and handles stationary and moving objects. In addition, the non-local-based fusion module only extracts key features from frame $F_{t-1}$. Then, extracted features are added to frame $F_{t}$. In comparison, the concatenation-based method directly fuses two frames of raw data without any selections. Therefore, the proposed method can enhance key features more efficiently.

We also compare the proposed method with popular 3D detectors on the NuScenes dataset. As shown in Table 4, the proposed method has the best performance among all detectors. In all categories, bus and barrier obtained the best performance, and the AP of the bus exceeds the second place AGO-Net by 2.7%. Even though the concatenation-based method uses 10 frames, the proposed method still surpasses them with respect to mean average precision compared with the multi-frame method 3D-VID, which is a pioneer of object detection methods based on point cloud videos. The mAP of the proposed method is 1.2% higher than that of the 3D-VID, which uses 30 frames.

Concatenating multiple frames can improve the detector’s performance, but it does not mean that the improvement has no limitations. Actually, concatenating more frames occupies many memory resources and exacerbates motion blur. Although 3D-VID deploys a deformable convolution on the premise of registration to alleviate motion blur, the unavoidable feature distortion and coarse feature interactions drag down the detector’s performance. The proposed MFFFNet can efficiently fuse two adjacent frames, and it shows good performance and great potential.

#### 4.3.2. Qualitative Analysis

In order to compare the detection results more clearly, we project point cloud scenes to the 2D plane. From the visualization results, we find that the proposed method is superior compared to backbone networks in two cases: 1. the objects that are far away from the sensor, as these objects only have a few valid points; 2. the objects that are occluded in one frame but are not occluded in another frame.

**Figure 9:**
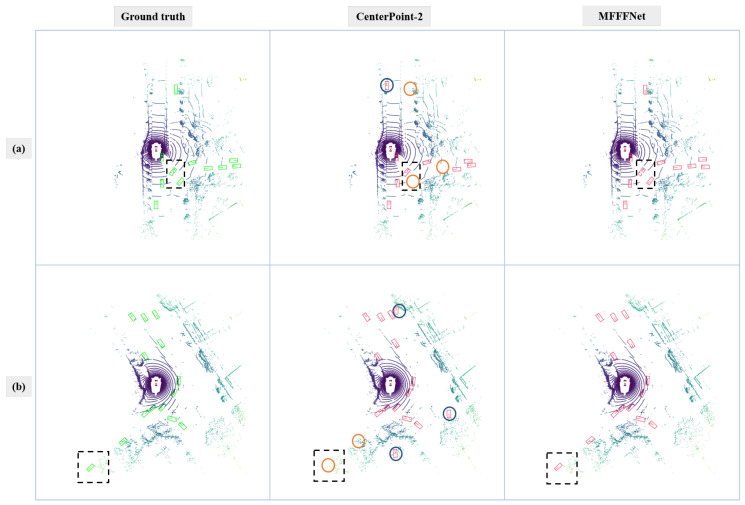
Comparison between MFFFNet and CenterPoint-2. Line (**a**,**b**) indicate two different scenes. The first column is the ground truth. The second and third columns are the detection results of the CenterPoint-2, and the MFFFNet, respectively. The green box represents the ground truth. The red box indicates the test results. The black dashed box indicates the areas that need to be focused on. Blue and orange circles indicate false positive and false negative results.

As shown in Figure 9, these two cases are marked with black dotted boxes. As shown in Figure 9a, a fan-shaped area is formed due to the obstruction of the upper left vehicle. In this area, two vehicles are turning. The proposed multi-frame detector successfully detects the two objects, while the CenterPoint-2 only detects one vehicle. In the occlusion area, objects’ point clouds are more sparse, which is challenging for the single-frame detector. In continuous frames, the occluded object may be exposed from the area, and scanned points of the object will become denser. Hence, the complementary information provided by other frames is beneficial for object detection. As shown in Figure 9b, the distant area was marked by the black box. The proposed method successfully detects the car, while CenterPoint-2 misses it.

**Figure 10:**
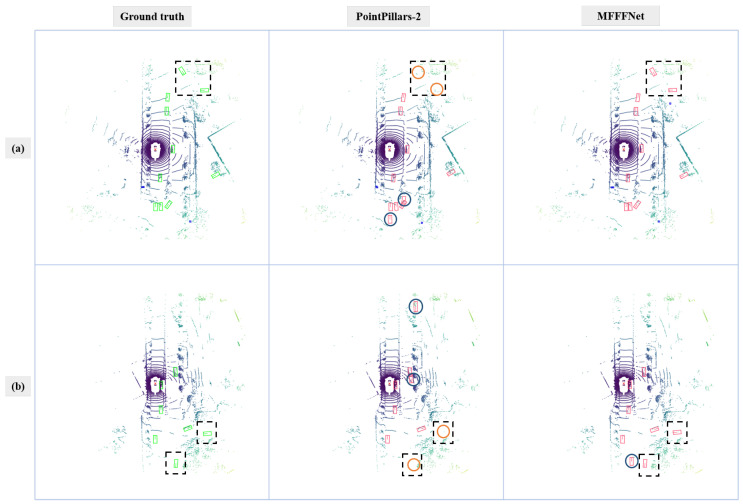
Comparison between MFFFNet and PointPillars-2. Line (**a**,**b**) indicate two different scenes. The first column is the ground truth. The second and third columns are the detection results of the CenterPoint-2, and the MFFFNet, respectively. The green box represents the ground truth. The red box indicates the test results. The black dashed box indicates the areas that need to be focused on. Blue and orange circles indicate false positive and false negative results.

A similar case occurs in Figure 10. Distant objects are missed by the PointPillars-2. The imaging performance of lidar decreases with distance, so there are few points in the distant area. These areas cannot provide sufficient information for the detector for recognizing objects. Thus, it is difficult for the two backbone networks to detect these sparse objects. The proposed method fuses the two frames at the feature level, which only fuses key features and ignores irrelevant features. Therefore, this method can utilize the information of two frames profoundly and efficiently.

#### 4.3.3. Ablation Studies

In this subsection, the effectiveness of each block is verified based on two backbone networks. First, the performance of PointPillars-2 and CenterPoint-2 is tested with the NuScenes dataset. Next, the proposed blocks are added to the fusion framework, which adopts PointPillars or CenterPoint to extract features of two frames. As shown in Table 5, the non-local-based fusion module and the index-nonlocal fusion module have the most contributions relative to the MFFFNet. In addition, TANet also brings considerable improvements for MFFFNet.

In summary, the non-local-based fusion method effectively strengthens key features by combining the two frames’ information. The index-nonlocal module significantly reduces the space resource occupation so that it can achieve the fusion of high-resolution feature maps. In addition, the TANet module can suppress background noise and enhance crucial information. In this manner, the distinction between foreground objects and background increased so that the possibility of objects being associated with background is reduced.

## 5. Conclusions

In this paper, we propose a non-local-based feature fusion method to fuse two frames of point cloud. The proposed method can handle both moving and static objects without external information-based registration. In order to reduce the resource consumption of the non-local module, we propose the index-nonlocal module. It improves the applicability of the fusion module and makes it possible to fuse the high-resolution feature map. In addition, considering that feature confusion may occur in fusion, we use the TANet module to enhance key information and suppress background noise. Based on the NuScenes dataset, the proposed method not only outperforms concatenation-based fusion methods but also exceeds the strong multi-frame detector, 3D-VID. In addition, the proposed method is verified based on both anchor-based and anchor-free methods. Experimental results indicate that the proposed method is effective and universally applicable.

## Figures and Tables

**Table 1:** Comparison between using 1 frame and 2 frames.

Method	Input	Fusion Mode	Registration	mAP
PointPillars-1	1-frame	/	/	32.7
PointPillars-2	2-frames	concatenate	Yes	34.7

Input represents the number of frames used in the experiment. Fusion mode indicates the fusion method used in the experiment. Registration indicates whether to perform the registration operation. mAP is the evaluation metric.

**Table 2:** Comparison between concatenation-based fusion method with and without registration.

Method	Input	Fusion Mode	Registration	mAP
PointPillars-2	2-frames	concatenate	Yes	34.7
PointPillars-2	2-frames	concatenate	No	32.6

**Table 3:** Comparison between MFFFNet, PointPillars, and CenterPoint.

Method	Input	Fusion mode	mAP	Car	Ped	Bus	Barrier	TC	Truck	Motor	Trailer	Bicycle	CV
PointPillars-2	2	concatenate	34.7	72.0	56.5	56.0	37.3	33.8	34.9	21.1	28.2	1.4	5.7
MFFFNet-PP	2	Non-local-based	38.6	73.6	60.9	60.4	44.1	39.0	36.2	24.2	33.4	1.8	12.5
improvement	/	/	+3.9	+1.6	+4.4	+4.4	+6.8	+5.2	+1.3	+3.1	+5.2	+0.4	+6.8
CenterPoint-2	2	concatenate	42.5	78.8	70.5	59.4	52.3	46.1	45.1	33.3	27.3	7.0	5.6
MFFFNet-CP	2	Non-local-based	46.6	79.7	74.1	64.9	56.5	51.6	46.8	36.5	32.5	14.1	8.9
improvement	/	/	+4.1	+0.9	+3.6	+5.5	+4.2	+5.5	+1.7	+3.2	+5.2	+7.1	+3.3

Ped and Motor are abbreviations of pedestrian and motorcycle, respectively. TC and CV represent the traffic cone and construction vehicle, respectively. Figures in the last 10 columns indicate the average precision (AP) of each class.

**Table 4:** Comparison between different methods on the NuScenes dataset.

Method	Input	Fusion mode	mAP	Car	Ped	Bus	Barrier	TC	Truck	Motor	Trailer	Bicycle	CV
MAIR [43]	10	concatenate	30.4	47.8	37.0	18.8	51.1	48.7	22.0	29.0	17.6	**24.5**	7.4
SARPNet [44]	10	concatenate	32.4	59.9	69.4	19.4	38.3	44.6	18.7	29.8	18.0	14.2	11.6
WYSWYG [24]	10	concatenate	35.4	80.0	66.9	54.1	34.5	27.9	35.8	18.5	28.5	0.0	7.5
GVNet [45]	10	concatenate	35.4	76.2	59.2	42.3	32.0	/	29.7	20.7	22.1	0.8	/
InfoFocus [46]	10	concatenate	36.4	77.6	61.7	50.5	43.4	33.4	35.4	25.2	25.6	2.5	8.3
BirdNet+ [47]	10	concatenate	36.5	67.3	48.6	48.4	51.5	18.9	44.4	31.4	32.3	10.0	12.4
3DSSD [48]	10	concatenate	42.7	81.2	70.2	61.4	47.9	31.1	47.2	36.0	30.5	8.6	12.6
AGO-Net [49]	10	concatenate	45.1	**81.5**	72.2	62.2	51.2	48.1	**50.1**	32.5	34.0	5.9	13.3
SSN [50]	10	concatenate	46.3	80.7	72.3	39.9	56.3	54.2	37.5	**43.7**	**43.9**	20.1	14.6
3D-VID [8]	10 × 3	ConvGRU	45.4	79.7	**76.5**	47.1	48.8	**58.8**	33.6	40.7	43.0	7.9	**18.1**
MFFFNet-CP	2	Non-local-based	**46.6**	79.7	74.1	**64.9**	**56.5**	51.6	46.8	36.5	32.5	14.1	8.9

Only the CenterPoint-based implementation (MFFFNet-CP) is selected for comparison, which is the most popular anchor-free detector.The maximum value in each column is marked in bold.

**Table 5:** Results of the ablation experiment.

Method	Input	Fusion Mode	mAP	Improvement
PointPillars-2	2	concatenate	34.7	/
PP + Non-local	2	Non -local -based	36.6	+1.9
PP + Non-local + TANet	2	Non -local -based	37.5	+0.9
PP + Non-local + TANet + Index-nonlocal	2	Non -local -based	38.6	+1.1
CenterPoint-2	2	concatenate	42.5	/
CP + Non-local	2	Non -local -based	43.9	+1.4
CP + Non-local + TANet	2	Non -local -based	44.7	+0.8
CP + Non-local + TANet + Index-nonlocal	2	Non -local -based	46.6	+1.9

PP and CP are the abbreviations for PointPillars and CenterPoint, respectively.

## Data Availability

Restrictions apply to the availability of these data. Data was obtained from Motional (formerly nuTonomy) and are available at https://www.nuscenes.org/nuscenes with the permission of Motional.
